# Zearalenone Promotes LPS-Induced Oxidative Stress, Endoplasmic Reticulum Stress, and Accelerates Bovine Mammary Epithelial Cell Apoptosis

**DOI:** 10.3390/ijms231810925

**Published:** 2022-09-18

**Authors:** Yurong Fu, Yongcheng Jin, Yue Tian, Hao Yu, Ruqi Wang, Huiyu Qi, Bo Feng, Jing Zhang

**Affiliations:** College of Animal Sciences, Jilin University, Changchun 130062, China

**Keywords:** zearalenone, lipopolysaccharide, oxidative stress, endoplasmic reticulum stress, apoptosis

## Abstract

Both zearalenone (ZEA) and lipopolysaccharide (LPS) can induce oxidative stress, and even apoptosis in bovine mammary epithelial cells (MAC-T), but not much attention has been given to the synergistic effect of ZEA and LPS. In this study, we treated MAC-T cells with different concentrations of LPS (1, 10, 50, and 100 μg/mL) and ZEA (5, 15, and 30 μM) to induce cell damage. Previous results show that MAC-T cell viability decreases with increasing LPS concentration. Meanwhile, 1 µg/mL LPS and ZEA were selected for combined treatment in subsequent studies. It was found that co-treatment with ZEA and LPS increases the accumulation of reactive oxygen species (ROS) and malondialdehyde (MDA), decreases mitochondrial membrane potential (MMP), and superoxide dismutase (SOD), and reduces glutathione (GSH). ZEA and LPS are found to activate endoplasmic reticulum (ER) stress by increasing the expression of glucose-regulated protein 78 kDa (GRP78), activating transcription factor 6 (ATF6) and C/EBP homologous protein (CHOP). It increases cell apoptosis by suppressing the expression of the anti-apoptotic protein B-cell lymphoma-2 (Bcl-2), indicated by up-regulation of Bcl2-associated X protein (Bax) and Cysteinyl aspartate-specific proteinases 3 (caspase-3) expression. The above results suggest that the synergistic effect of ZEA and LPS aggravate cytotoxicity.

## 1. Introduction

Mycotoxins are frequently found contaminants in agricultural products such as corn and other grains used for feed. Zearalenone (ZEA), one of the most important mycotoxins, is formed by many species of the genus Fusarium [1]. The contamination of food and feed with ZEA has adverse health and economic effects [2]. In addition, ZEA is also shown to have hepatotoxicity, hematological toxicity, immunotoxicity, and genotoxicity [3,4,5,6]. For dairy cows, complex rations of ruminants consisting of forage, concentrate, and silage can be a source of multiple mycotoxins [7], and contaminated animal feed can lead to reduced productivity and health impairments in dairy cows. Corn silage is associated with the production of mycotoxins, and although some mycotoxins can be degraded by the rumen microbiota of ruminants, subclinical health problems associated with mycotoxins are reported in high-yielding dairy cows [8]. The rumen microbiota can degrade certain mycotoxins into less toxic substances, thus, exerting a protective effect [9]. However, in the case of ZEA, microbiota-mediated metabolism in the rumen may not lead to detoxification [10]. Although the rumen is considered a barrier against mycotoxins, some studies suggest that mycotoxins may be carried into milk. Our previous study shows that ZEA causes endoplasmic reticulum(ER) stress apoptosis in bovine mammary epithelial cells [11]. Related studies report that, among other mycotoxins (deoxynivalenol or ochratoxin A), co-action with lipopolysaccharide(LPS)increases inflammation in the body [12,13]. A study of gastrointestinal Fusarium toxins in endotoxemic pigs shows that the gastrointestinal metabolism of ZEA is significantly affected in endotoxemic pigs [14].

Intramammary infusion of Gram-negative LPS was previously used to induce inflammation in the mammary glands of cows and mice [15]. Trichothecenes mycotoxins are shown to activate caspase-1 in human macrophages, and further induce LPS-triggered cells to secrete IL-1β and IL-18 [16]. As a key factor in various intestinal inflammations, LPS can disrupt epithelial barrier function. The combined enterotoxicity of LPS and deoxynivalenol (DON) demonstrates that non-toxic doses of DON aggravate LPS-induced cellular inflammatory responses, further suggesting that the ingestion of low-dose mycotoxins may exacerbate the effects of enteric pathogens on the body [17].

Then, if passive feeding of ZEA increases LPS, what is the effect of co-acting these two toxins on cow mammary epithelial cells? No one has seriously discussed this idea yet. If the content of ZEA in the feed here is in the standard category, will the coexistence with LPS accelerate the toxic effect of ZEA? It is necessary to discuss the combined toxicity of ZEA and endotoxins to provide necessary insights for safe feed production.

## 2. Results

### 2.1. ZEA and LPS Reduce MAC-T Cells Viability

**Figure 1:**
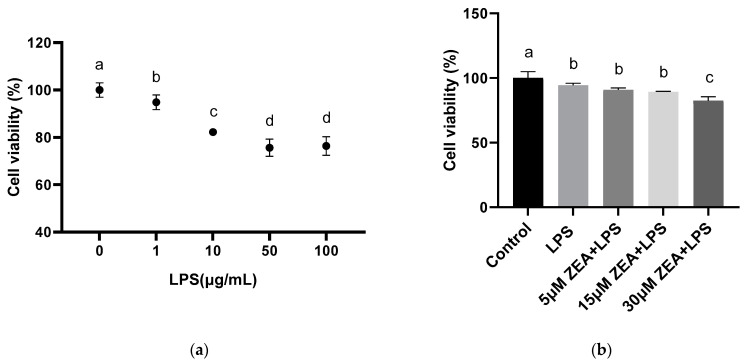
Zearalenone (ZEA) and lipopolysaccharide (LPS) reduce MAC-T cells viability. (**a**) Different concentrations (1, 10, 50, and 100 μg/mL) of LPS treated MAC-T cells for 24 h to detect cell viability. (**b**) MAC-T cells co-treated with 1 μg/mL LPS and 5, 15, and 30 μM ZEA for 24 h to detect cell viability. Each experiment was repeated 3 times. All values are expressed as mean ± SD (*n* = 3). Different letters a, b, and c indicate *p* < 0.05.

To study the effects of LPS and ZEA on bovine mammary epithelial cells (MAC-T), MAC-T cells were treated with different concentrations of LPS and ZEA. The results of this study show that with different concentrations of LPS (0, 1, 10, 50, and 100 μg/mL), compared with the control group, the cell viability is significantly decreased at the concentrations of 1, 10, 50, and 100 μg/mL (*p* < 0.05, Figure 1a). We subsequently selected 1 μg/mL of LPS to combine with ZEA (5, 15, and 30 μM). The results show that, compared with the control group, the LPS group, 5 μM ZEA + LPS group, 15 μM ZEA + LPS group, and 30 μM ZEA + LPS group are all significantly decreased (*p* < 0.05, Figure 1b). Compared with the LPS group, the 30 μM ZEA + LPS group is significantly lower (*p* < 0.05, Figure 1b).

### 2.2. ZEA and LPS Increase LDH in MAC-T Cells

**Figure 2:**
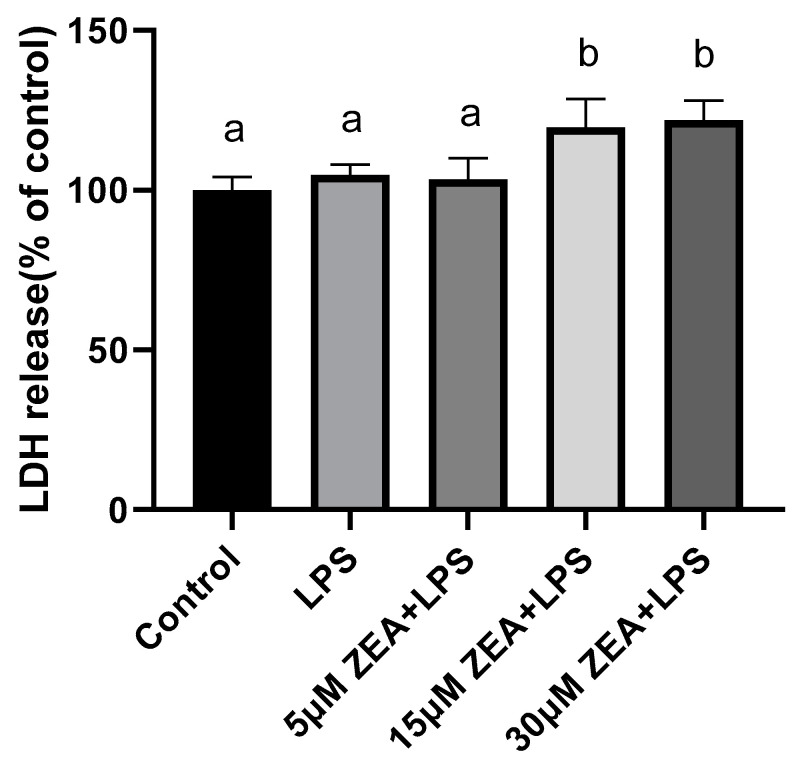
ZEA and LPS increase LDH in MAC-T cells. MAC-T cells were treated with 1 μg/mL LPS and 5, 15, and 30 μM ZEA for 24 h to detect the LDH content. Each experiment was repeated 3 times. All values are expressed as mean ± SD (*n* = 3). Changes in cell viability were determined by independent samples *t*-test. Different letters a and b indicate *p* < 0.05.

The results of lactate dehydrogenase (LDH) show that, compared with the control group, there is a significant difference between the 15 μM ZEA + LPS group and the 30 μM ZEA + LPS group (*p* < 0.05, Figure 2), and there is no difference with the 5 μM ZEA + LPS group. Compared with the LPS group, there is a significant difference between the 15 μM ZEA + LPS group and the 30 μM ZEA + LPS group (*p* < 0.05, Figure 2), but there is no difference with the 5 μM ZEA + LPS group. In conclusion, the 30 μM ZEA + LPS group has a greater effect on cell viability and LDH.

### 2.3. Combination of ZEA and LPS Causes the Oxidative Stress Response of MAC-T Cells

**Figure 3:**
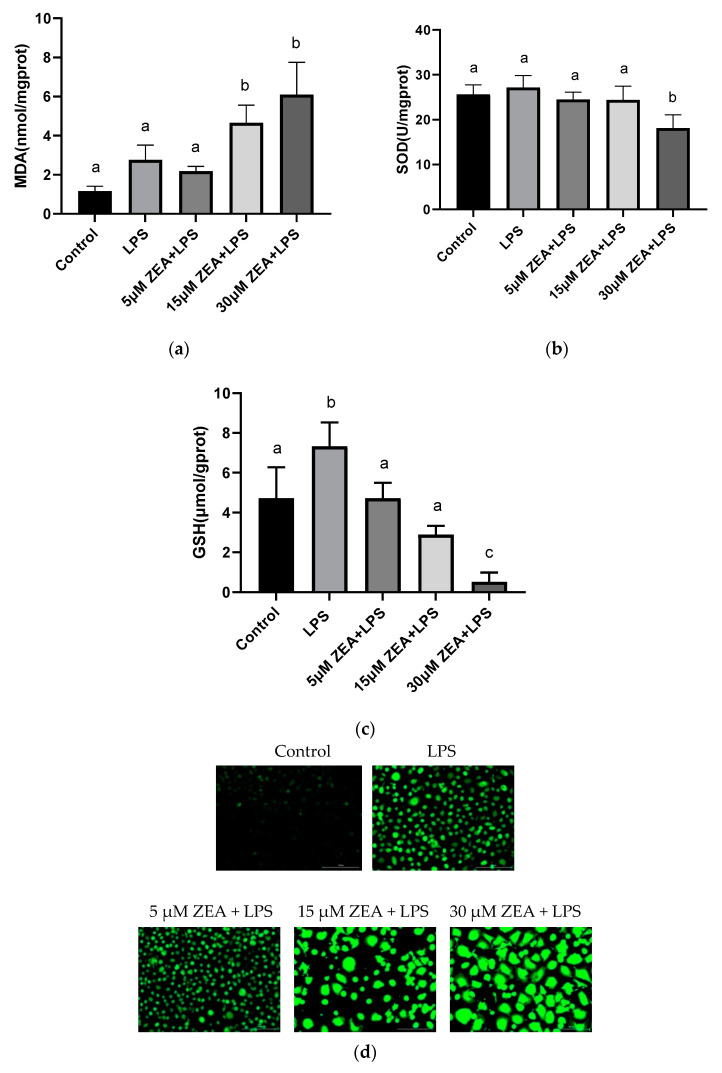

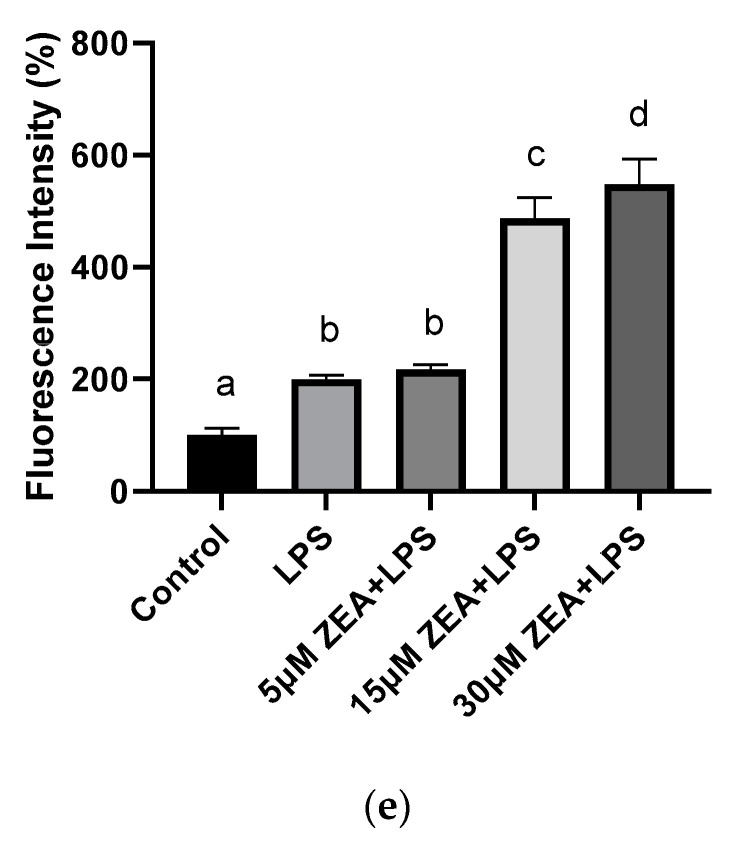
Combination of ZEA and LPS causes the oxidative stress response of MAC-T cells. (**a**–**c**) MDA, SOD, and GSH levels are detected after MAC-T cells were treated with 1 μg/mL LPS and 5, 15, and 30 μM ZEA for 24 h. (**d**) Untreated control or LPS and ZEA-treated MAC-T cells were stained with DCFH-DA probe. (**e**) Fluorescence intensity data based on the images in Figure 3d. Each experiment was repeated 3 times. All values are expressed as mean ± SD (*n* = 3). Different letters a, b, c, and d indicate *p* < 0.05. Scale bar = 200 μm.

The data show that, compared with the control group, both the 15 μM ZEA + LPS group and the 30 μM ZEA + LPS group significantly increase the malondialdehyde (MDA) content of MAC-T cells (*p* < 0.05) (Figure 3a), and the 30 μM ZEA + LPS group significantly decreases MAC-T cells superoxide dismutase (SOD) and reduces glutathione (GSH) activities of cells (*p* < 0.05) (Figure 3b,c). Reactive oxygen species (ROS) is a direct evaluation index to detect the content of oxidative free radicals. As presented in Figure 3d,e, compared with the control group, the ROS of the other four groups are significantly increased (*p* < 0.05). Compared with the LPS group, ROS are significantly increased in the 15 μM ZEA + LPS group and the 30 μM ZEA + LPS group (*p* < 0.05). These data reveal that the oxidative stress leads to the accumulation of the ROS in the cells.

### 2.4. The Combination of ZEA and LPS Reduces the Mitochondrial Membrane Potential of MAC-T Cells

**Figure 4:**
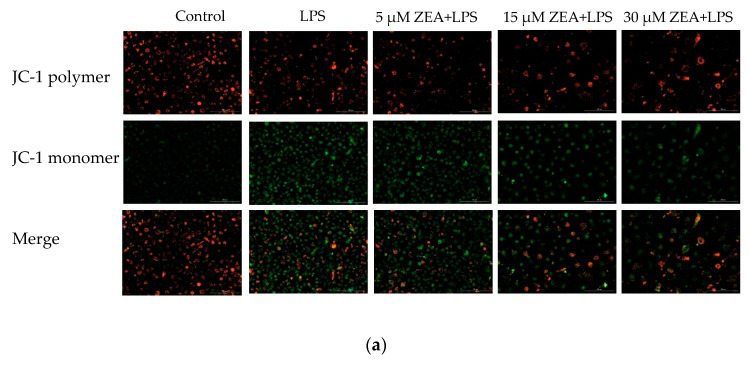

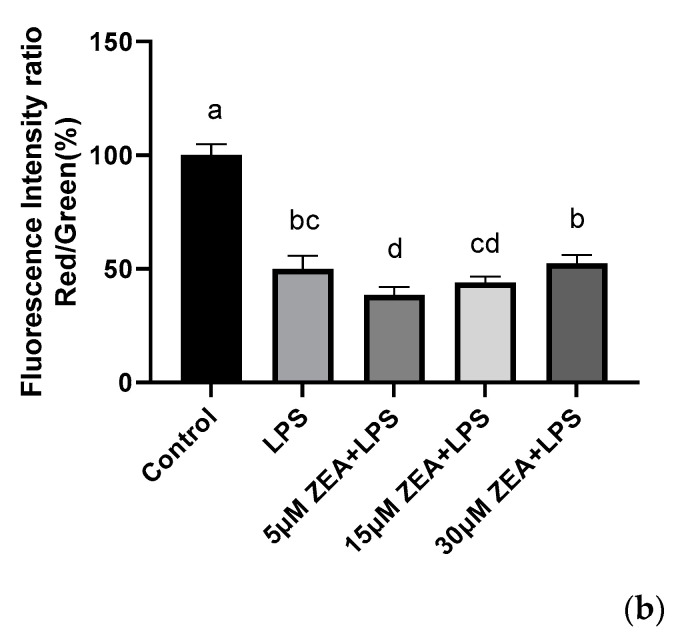
The combination of ZEA and LPS reduces the mitochondrial membrane potential of MAC-T cells. (**a**) Changes in mitochondrial membrane potential after treatment of MAC-T cells with LPS and ZEA for 24 h. (**b**) Plotted from the ratio of fluorescence intensities in Figure 4a. Each experiment was repeated 3 times. All values are expressed as mean ± SD (*n* = 3). Different letters a, b, c, and d indicate *p* < 0.05. Scale bar = 200 μm.

Next, we measured changes in mitochondrial membrane potential (MMP) in MAC-T cells under different treatments using the JC-1 fluorescent probe. The results of this study show that, compared with the control group, MMP is significantly decreased in the LPS group (*p* < 0.05, Figure 4), and the combined treatment of ZEA and LPS is also significantly decreased (*p* < 0.05, Figure 4). Compared with the LPS group, the 5 μM ZEA + LPS group is significantly decreased (*p* < 0.05, Figure 4), and the 15 and 30 μM ZEA + LPS groups are not. This indicates that the combination of LPS and ZEA has a significant effect on mitochondrial damage.

### 2.5. Combined Treatment of ZEA and LPS Induces ER Stress in MAC-T Cells

**Figure 5:**
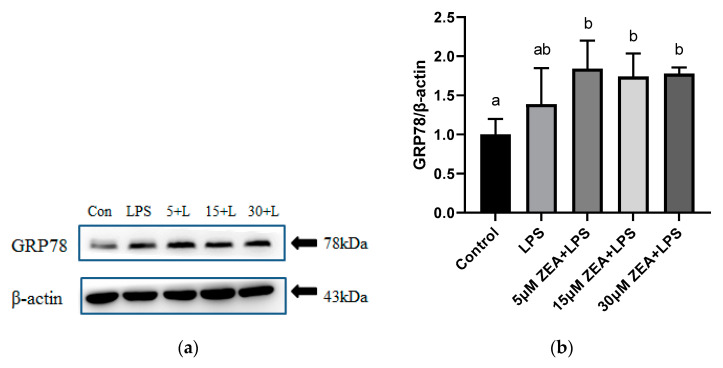

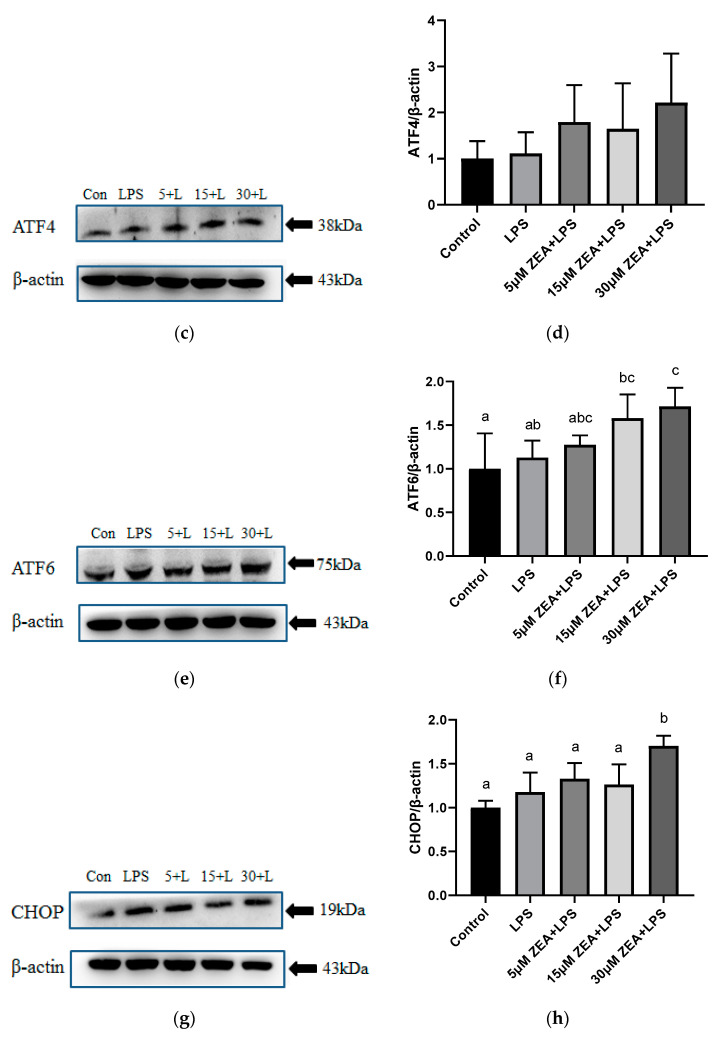
Combined treatment of ZEA and LPS induces ER stress in MAC-T cells. Relative protein expression of GRP78, ATF6, ATF4, and CHOP in MAC-T cells after treatment with 1 μg/mL LPS and 5, 15, and 30 μM ZEA for 24 h. All values are expressed as mean ± SD (*n* = 3). Different letters a, b, and c indicate *p* < 0.05. (**a**,**b**) Protein expression of GRP78 determined by western blotting in MAC-T cells; (**c**,**d**) Protein expression of ATF4 determined by western blotting in MAC-T cells;(**e**,**f**) Protein expression of ATF6 determined by western blotting in MAC-T cells; (**g**,**h**) Protein expression of CHOP determined by western blotting in MAC-T cells.

As shown in Figure 5a,b, glucose-regulated protein 78 kDa (GRP78) protein levels are increased in the 5 μM ZEA + LPS group, 15 μM ZEA + LPS group, and 30 μM ZEA + LPS group, compared with the control group (*p* < 0.05). Compared with the control group, the activating transcription factor 4(ATF4) protein levels of the other four groups are not significantly different(Figure 5c,d). Compared with the control group, the activating transcription factor 6(ATF6) protein level is increased in the 15 μM ZEA + LPS group and the 30 μM ZEA + LPS group (*p* < 0.05, Figure 5e,f). Compared with the LPS group, the ATF6 protein level is significantly increased in the 30 μM ZEA + LPS group (*p* < 0.05, Figure 5e,f). In the detection of C/EBP homologous protein (CHOP), compared with the control group, the level of CHOP protein in the 30 μM ZEA + LPS group is increased (*p* < 0.05, Figure 5g,h). Compared with the LPS group, the CHOP protein level in the 30 μM ZEA + LPS group is increased (*p* < 0.05, Figure 5g,h).

### 2.6. The Combined Effect of ZEA and LPS Accelerates the Apoptosis of MAC-T Cells

**Figure 6:**
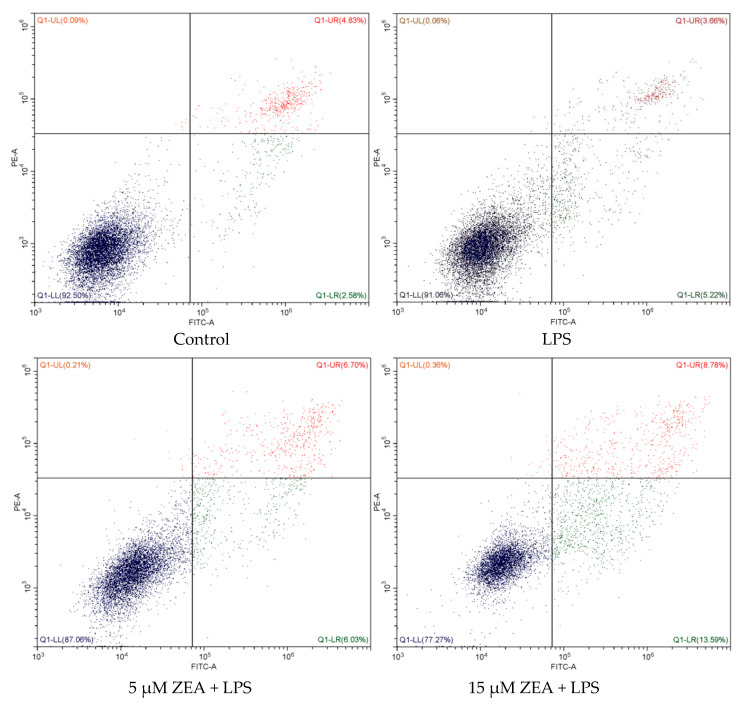

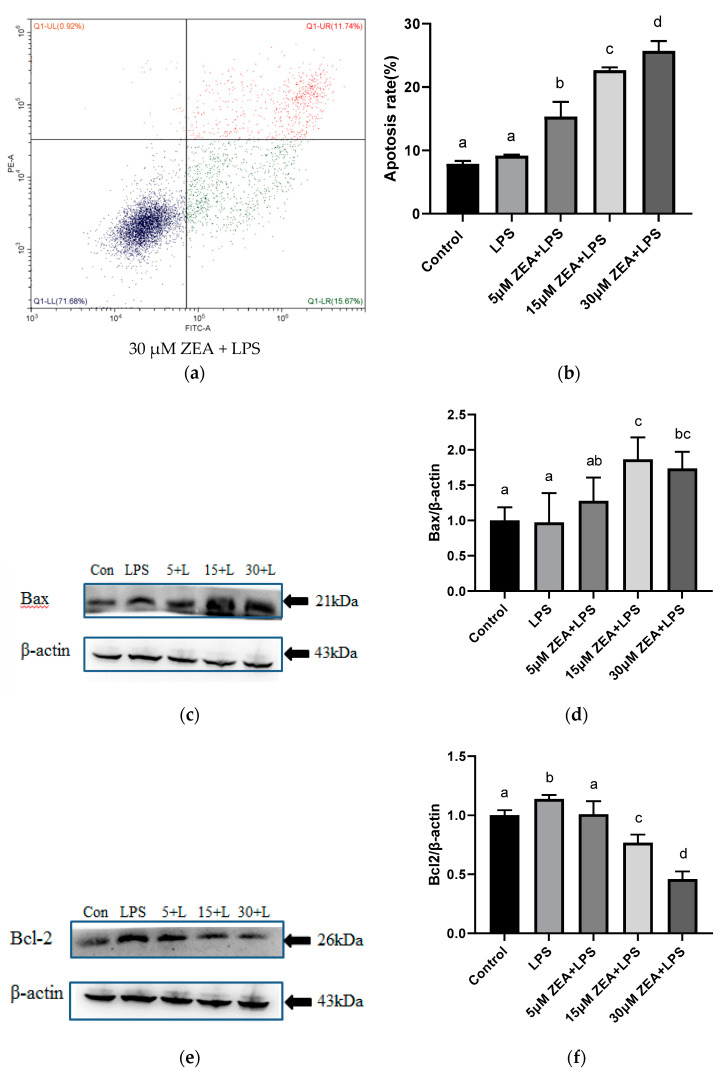

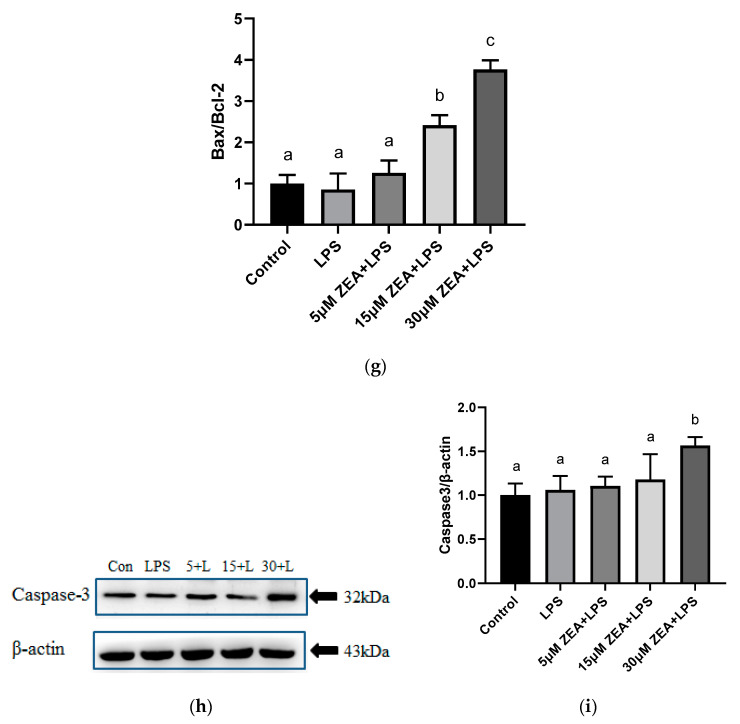
The combined effect of ZEA and LPS accelerates the apoptosis of MAC-T cells. (**a**,**b**) Effects of 1 μg/mL LPS and 5, 15, and 30 μM ZEA on apoptosis in MAC-T cells after 24 h of treatment using flow cytometry. (**c**–**f**) Relative protein expression of Bax and Bcl-2. (**g**) The ratio of Bax to Bcl-2 protein expression. (**h**,**i**) Relative protein expression of caspase-3. All values are expressed as mean ± SD (*n* = 3). Different letters a, b, and c indicate *p* < 0.05.

Meantime, this study examined the effects of ZEA and LPS on the apoptosis of MAC-T cells. As shown in Figure 6a,b, the results of flow cytometry to detect cell apoptosis show that, compared with the control group, there are no significant differences in the LPS group, but with the increase in ZEA concentration, the combination of ZEA and LPS-treated cell apoptosis rate increases significantly. As shown in Figure 6c,d, compared with the control group, the Bax protein level is significantly increased in the 15 μM ZEA + LPS group and the 30 μM ZEA + LPS group (*p* < 0.05). Compared with LPS, Bax protein levels are significantly increased in 15 μM ZEA + LPS group and 30 μM ZEA + LPS group (*p* < 0.05). In Figure 6e,f, compared with the control group, the Bcl-2 protein level in the LPS group is significantly increased (*p* < 0.05), and the Bcl-2 protein level in the 15 μM ZEA + LPS group and the 30 μM ZEA + LPS group is significantly decreased (*p* < 0.05). Compared with the LPS group, the concentration of ZEA decreases significantly (*p* < 0.05). As shown in Figure 6g, compared with the control group, the Bax/Bcl-2 ratio in the 15 μM ZEA + LPS group and the 30 μM ZEA + LPS group is increased (*p* < 0.05). Compared with the LPS group, the Bax/Bcl-2 ratio of the 15 μM ZEA + LPS group and the 30 μM ZEA + LPS group increases (*p* < 0.05). As shown in Figure 6h,i, compared with the control group, the caspase-3 protein level in the 30 μM ZEA + LPS group is significantly increased (*p* < 0.05). Compared with LPS, the protein level of caspase-3 in the 30 μM ZEA + LPS group is significantly increased (*p* < 0.05).

## 3. Discussion

Studies show that LPS can synergistically enhance the toxicity of trichothecenes mycotoxins. In a combined enterotoxicity study, non-toxic doses of DON aggravates LPS-induced intestinal inflammation and tight junction disorders, by activating the NF–κB signaling pathway and autophagy-related protein LC3B [17]. We speculate that the combination of ZEA and LPS has a synergistic effect on bovine mammary epithelial cells, so it is necessary to explore this issue. Our study shows that cell viability decreases with increasing concentrations of LPS (0–100 μg/mL). When the concentration is 1 μg/mL, it is significantly reduced compared with the control group. We then used LPS 1 μg/mL in combination with ZEA at 5, 15, and 30 μM. As the concentration of ZEA increases, the combination of ZEA and LPS at 30 μM significantly reduces cell viability compared to the LPS-treated group. LDH is an enzyme that exists stably in the cytoplasm, and generally only exists in cells. LDH is a membrane-bound enzyme, and the leakage of LDH into the medium is usually associated with membrane damage. When the plasma membrane ruptures, LDH is rapidly released outside the cell, so it is a common tool for assessing toxicity in vitro [18,19]. Our results show that the 15 μM ZEA+LPS and 30 μM ZEA + LPS groups are significantly different from the control and LPS-treated groups. In conclusion, the combination of ZEA and LPS inhibits cell viability and LDH release, and the toxic effect on cells is deepened.

During oxidative stress, the oxidative damage caused by free radicals has a damaging effect on most biological macromolecules. It is reported that an imbalance between increased ROS production and decreased antioxidant defenses during parturition increases oxidative stress, and may lead to perinatal disease in dairy cows [20]. In studies on human bronchial epithelial cells, it is reported that ROS may play a key role in ZEA-induced cell death [21]. The accumulation of ROS is a direct manifestation of the detection of oxidative free radical content. Our results show that the accumulation of ROS is more significant in the combined treatment of 15 μM ZEA + LPS and 30 μM ZEA + LPS compared to the LPS-treated group. Our previous study of ROS levels in MAC-T cells 24 h after 30 μM ZEA treatment shows that the ROS accumulation in the 30μM ZEA group is extremely significantly increased compared with the control group [11]. Meanwhile, other studies show that in MAC-T cells, ROS are significantly increased after treatment with LPS (100 µg/mL) for 24 h [22].

Mitochondria are the site of cellular energy metabolism, and play a key role in activating apoptosis in mammalian cells [23]. ROS trigger a decrease in MMP and an increase in the ratio of Bax/Bcl2 leading to the mitochondria-mediated pathway involved in apoptosis [24]. Next, we measured changes in mitochondrial membrane potential in MAC-T cells under different treatments using the JC-1 fluorescent probe. The results of this study show that the MMP of LPS 1 μg/mL is significantly decreased compared with the control group. MMP also shows a clear downward trend after combination with ZEA of 5, 15, and 30 μM. This suggests that the combination of LPS and ZEA has a significant effect on mitochondrial damage. ZEA induces oxidative stress, which further leads to mitochondrial dysfunction and DNA damage in early embryonic development in pigs [25]. ZEA induction induces an increase in intracellular ROS and a decrease in MMP in HEK293 cells [26].

There is growing evidence for the interrelationship between ER stress and ROS and mediators of redox signaling [27,28]. The endoplasmic reticulum (ER) is sensitive to oxidative stress, and also plays a critical role in oxidative stress-induced damage, when the perturbation of ER function induces cellular damage and leads to apoptosis [29]. Our results show that the combined ZEA + LPS treatment group increases the protein levels of GRP78, and CHOP compared to the control group. Meanwhile, the 15 μM ZEA + LPS and 30 μM ZEA + LPS treatment groups increase the protein level of ATF6. There are no significant differences in ATF4 protein. According to other research in the literature, LPS can induce testicular injury, apoptosis, ERS, and inflammatory responses in mouse spermatogonial stem cells (SSCs), in which ERS-related apoptotic proteins are activated and ERS gene expression is significantly up-regulated [30]. ZEA-induced apoptosis in human leukemia HL-60 and U937 cells activates mitochondrial release of cytochrome c through a reduction in mitochondrial transmembrane potential, production of reactive oxygen species, and the induction of endoplasmic reticulum stress [31].

The pro-apoptotic effect of LPS is demonstrated in Caco-2 human intestinal epithelial cells and bovine glomerular endothelial cells [26,32]. The bovine mammary epithelial cells treated with 100 ng/mL LPS are found to reduce cell viability, attenuate the expression of Bcl-2 mRNA, and significantly increase the expression of Bax mRNA [33]. The effects of LPS and ZEA on the apoptosis of MAC-T cells are determined. In terms of protein levels, the 15 uM ZEA + LPS group and the 30 μM ZEA + LPS group increase Bax protein levels and decrease Bcl-2 protein levels compared with the LPS alone group. In terms of Bax/Bcl-2 ratio, the 15 μM ZEA+LPS group and the 30 μM ZEA + LPS group are significantly increased compared with the LPS group. The results of flow cytometry to detect the apoptosis rate also show that the combination of ZEA and LPS treatment of MAC-T cells, compared with the LPS group treatment, increases the apoptosis rate. In our previous study, the results show that 30 μM ZEA treatment of MAC-T cells for 24 h induces apoptosis [11].

In addition to common reproductive system problems, ZEA has the potential for adverse gastrointestinal effects and carcinogenic potential [34,35,36]. According to our previous study on mycotoxins, polydatin alleviates ER stress and apoptosis of MAC-T cells caused by ZEA [37]. Other studies show that curcumin inhibits ZEA-induced apoptosis and oxidative stress in Leydig cells [38]. Overall, compound toxin effects deserve more attention than either LPS alone or the presence of potential ZEA in the feed. Most studies focus on the harm of single mycotoxins or a combination of mycotoxins, with little consideration of how other toxins work synergistically to affect cells. In future studies, it is necessary for us to re-examine the combined effects of these toxins at low concentrations, which are harmful to the dairy industry. Regarding the synergistic effect of ZEA and LPS, whether effective plant extracts or compounds can be found to reduce these adverse reactions is the question of our follow-up research.

## 4. Materials and Methods

### 4.1. Chemicals and Reagents

The ZEA (purity > 99%, Z2125), dimethyl sulfoxide (DMSO), and LPS (Escherichia coli 0111: B4) were purchased from Sigma Aldrich (St Louis, MO, USA). ZEA was dissolved in DMSO and stored at −20 °C. Fetal bovine serum (FBS) was purchased from Gibco (Gaithersburg, MD, USA) and high-glucose DMEM was purchased from HyClone (Logan, UT, USA). Cell Counting Kit-8 (CCK-8) was purchased from Dojindo Laboratories (Kumamoto, Japan). Kits for measuring ROS and for apoptosis detection were purchased from Beyotime Biotechnology (Shanghai, China). The kit for detecting lactate dehydrogenase (LDH) activity, the malondialdehyde (MDA) assay kit, superoxide dismutase (SOD) assay kit, and reduced glutathione (GSH) assay kit were purchased from Nanjing Jiancheng Bioengineering Institute (Nanjing, China). For Western blotting analysis, RIPA buffer (high) for total protein extraction was purchased from Beijing Solarbio Science & Technology Co., Ltd. (Beijing, China), while those for GRP78, CHOP, Bax, and Bcl-2 were purchased from Proteintech (Wuhan, China) and antibodies against caspase-3 was purchased from Abcam (Cambridge, MA, USA). Antibodies against ATF4 and ATF6 were purchased from Bioss (Beijing, China). Antibodies against β-actin was purchased from Affinity Biosciences (Cincinnati, OH, USA).

### 4.2. Cell Culture

The bovine mammary epithelial cell line MAC-T was kindly provided by Professor Hong Gu Lee (Konkuk University, Seoul, Korea). MAC-T cells were kept in high-glucose DMEM containing 10% FBS, 1% penicillin-streptomycin, 1 μg/mL hydrocortisone, and 5 μg/mL insulin for *in vitro* investigations, and they were incubated at 37 °C with 5% CO_2_.

### 4.3. Cell Treatment

LPS was dissolved in PBS at a concentration of 5 mg/mL for storage and diluted to different concentrations in DMEM for cell treatments. ZEA was dissolved in DMSO at a concentration of 100 mM for storage and diluted to the specific concentrations needed. The ZEA concentrations used to treat MAC-T cells were 5, 15, and 30 μM, and the LPS were 1, 10, 50, and 100 μg/mL.

### 4.4. Cell Viability Assay

Cell viability was measured using the CCK-8 kit following the manufacturer’s instructions. When cells reached 70–80% confluency, they were treated with different concentrations of LPS (1, 10, 50, and 100 μg/mL) and ZEA (5, 15, and 30 μM) for 24 h. Then, the cell viability was detected, a total of 10 μL of CCK-8 reagent was added to each well, and the cells were incubated at 37 °C for 1.5 h. Then, cell viability was measured using a microplate reader. Cell viability was calculated from the absorbance value (OD) of each well, measured at 450 nm.

### 4.5. Lactate Dehydrogenase Assay

LDH levels were detected using an LDH kit. LPS 1 μg/mL was seeded in 6-well plates in combination with different concentrations of ZEA (5, 15, and 30 μM). Cell culture medium was collected after 24 h. Blank wells, standard wells, assay wells, and control wells were set. Reagents and samples were added according to the kit instructions. Measured using a microplate reader and calculated from the absorbance value (OD) of each well, measured at 450 nm.

### 4.6. Antioxidant Enzymes Activities and Malondialdehyde (MDA) Levels

Levels in MAC-T cells were measured using MDA, GSH, and SOD kits according to the instructions (Nanjing Jiancheng Institute of Biotechnology, Nanjing, China). For sample processing, the treated cells were aspirated, the supernatant was also aspirated, and the cells were scraped directly with the cell-scraping method. Protein concentration was detected using the BCA Protein Assay Kit. Reagents were added one by one according to the kit instructions, and microplate reader detection was performed.

### 4.7. Measurement of ROS Production

ROS detection was performed using the fluorescent probe DCFH-DA. DCFH-DA was diluted 1:1000 in serum-free medium to a final concentration of 10 μmol/L. The cell culture medium was removed and an appropriate volume of diluted DCFH-DA was added. The volume to be added was sufficient to cover the cells. Usually, no less than 1 mL of diluted DCFH-DA is added to one well of a 6-well plate. Cells were incubated in a 37 °C incubator for 20 min. The cells were washed 3 times with serum-free cell culture medium to sufficiently remove DCFH-DA that did not enter the cells. Fluorescence intensity was measured using BioTek’s CYTATION 5 Imaging reader (Winooski, VT, USA).

### 4.8. Mitochondrial Membrane Potential Assay

Using JC-1 as a fluorescent probe, the MMP of cells was detected. The amount of JC-1 staining working solution required for each well of the 6-well plate is 1 mL, the culture solution was removed by suction, the cells are washed once with PBS, and 1 mL of cell culture solution was added. The cell culture medium may contain serum and phenol red. Adding 1 mL of JC-1 staining working solution and mixing well. Then, cells were placed in a cell incubator at 37 °C for 20 min. During the incubation period, an appropriate amount of JC-1 staining buffer was prepared in an ice bath. After incubation at 37 °C, the supernatant was removed by suction and washed twice with JC-1 staining buffer (1×). Adding 2 mL of cell culture medium, which may have contain serum and phenol red. Fluorescence intensity was measured using BioTek’s CYTATION 5 Imaging reader.

### 4.9. Apoptosis Detection by Flow Cytometry

The cell culture medium was collected, cells were trypsinized, and centrifuged with the collected cell culture medium. It was centrifuged at 1000× *g* for 5 min. The cell pellet was collected after discarding the supernatant, gently resuspended it in 1× PBS, and performed a cell count. Subsequently, 5 × 10^4^/1 × 10^5^ cells were centrifuged at 1000× *g* for 5 min. After the supernatant was discarded, adding 195 μL of AnnexinV-FITC binding solution into cells, and gently resuspended them. Then, adding5 μL of AnnexinV-FITC and mixing gently. A total of 10 μL of PI staining solution was added and mixed gently. Cells were incubated at room temperature protected from light for 10-20 minutes. It was then placed in an ice bath protected from light and awaited detection.

### 4.10. Western Blotting

Total protein was extracted from cells using RIPA buffer (high), and the concentration of the extracted protein was determined by the BCA method. After determining the extracted protein concentration, each protein sample was mixed with loading buffer and denatured at 100 °C for 5 min. Then, 12% sodium dodecyl sulfate polyacrylamide (SDS–PAGE) was performed. Samples containing 20 µg of protein were added to each well of an SDS–PAGE gel, then transferred to PVDF membrane. Membranes were washed with Tris-buffered saline containing Tween (TBST) and mixed with appropriate rabbit primary antibodies specific for GRP78, ATF4, ATF6, CHOP, caspase-3, Bcl-2, or Bax at 4 °C. These were incubated together overnight. After washing four times with TBST, the immunoblot was incubated with horseradish peroxidase-conjugated goat anti-rabbit immunoglobulin G (IgG) secondary antibody for 2 h at room temperature. Finally, the bands density of proteins were quantified by Image J.

### 4.11. Statistical Analyses

The data between groups was analyzed using SPSS statistical software (version 19.0). All experiments were repeated with 3 independent replicates. Two treatment effects were evaluated using one-way ANOVA followed by Duncan’s multiple range test. Data are expressed as the mean ± SD. Differences of *p* < 0.05 were considered statistically significant.

## 5. Conclusions

The results of this study show that the synergistic effect of ZEA and LPS decreases cell viability, increases accumulation of LDH and ROS, decreases MMP, leads to endoplasmic reticulum stress, and accelerates apoptosis.

## Data Availability

Not applicable.
